# The Dietary Intervention of Transgenic Low-Gliadin Wheat Bread in Patients with Non-Celiac Gluten Sensitivity (NCGS) Showed No Differences with Gluten Free Diet (GFD) but Provides Better Gut Microbiota Profile

**DOI:** 10.3390/nu10121964

**Published:** 2018-12-12

**Authors:** Carmen Haro, Myriam Villatoro, Luis Vaquero, Jorge Pastor, María J. Giménez, Carmen V. Ozuna, Susana Sánchez-León, María D. García-Molina, Verónica Segura, Isabel Comino, Carolina Sousa, Santiago Vivas, Blanca B. Landa, Francisco Barro

**Affiliations:** 1 Departamento de Protección de Cultivos, Instituto de Agricultura Sostenible (IAS-CSIC), 14004 Córdoba, Spain; hamac.7@hotmail.com (C.H.); blanca.landa@ias.csic.es (B.B.L.); 2 Departamento de Mejora Genética Vegetal, Instituto de Agricultura Sostenible (IAS-CSIC), 14004 Córdoba, Spain; myriamvillatoro@gmail.com (M.V.); mjga06@ias.csic.es (M.J.G.); carpirinha32@hotmail.com (C.V.O.); ssanchez@ias.csic.es (S.S.-L.); mdgarcia@ias.csic.es (M.D.G.-M.); 3 Departamento de Gastroenterología, Hospital de León, Instituto de Biomedicina, Universidad de León, 24071 León, Spain; luisvaqueroayala@gmail.com (L.V.); svivasa@gmail.com (S.V.); 4 Novapan, S.L., C/Chopo, 68-70, 50171 La Puebla de Alfinden, Zaragoza, Spain; jorge-pastor@panishop.com; 5 Departamento de Microbiología y Parasitología, Facultad de Farmacia, Universidad de Sevilla, 41004 Seville, Spain; vsegura@us.es (V.S.); icomino@us.es (I.C.); csoumar@us.es (C.S.)

**Keywords:** NCGS, gluten, gut-microbiota, next-generation sequencing, digestive disorder

## Abstract

The study evaluated the symptoms, acceptance, and digestibility of bread made from transgenic low-gliadin wheat, in comparison with gluten free bread, in Non-coeliac gluten sensitivity (NCGS) patients, considering clinical/sensory parameters and gut microbiota composition. This study was performed in two phases of seven days each, comprising a basal phase with gluten free bread and an E82 phase with low-gliadin bread. Gastrointestinal clinical symptoms were evaluated using the Gastrointestinal Symptom Rating Scale (GSRS) questionnaire, and stool samples were collected for gluten immunogenic peptides (GIP) determination and the extraction of gut microbial DNA. For the basal and E82 phases, seven and five patients, respectively, showed undetectable GIPs content. The bacterial 16S rRNA gene V1-V2 hypervariable regions were sequenced using the Illumina MiSeq platform and downstream analysis was done using a Quantitative Insights into Microbial Ecology (QIIME) pipeline. No significant differences in the GSRS questionnaires were observed between the two phases. However, we observed a significantly lower abundance of some gut genera *Oscillospira*, *Dorea*, *Blautia*, *Bacteroides*, *Coprococcus*, and *Collinsella,* and a significantly higher abundance of *Roseburia* and *Faecalibacterium* genera during the E82 phase compared with the basal phase. The consumption of low-gliadin bread E82 by NCGS subjects induced potentially positive changes in the gut microbiota composition, increasing the butyrate-producing bacteria and favoring a microbial profile that is suggested to have a key role in the maintenance or improvement of gut permeability.

## 1. Introduction

Globally, wheat is the cereal most used for bread. Although only 35% of all wheat production is consumed by humans, it dominates the international grain trade as 110 of the 250 million tons of traded grain is wheat (http://faostat3.fao.org/). There is an increasing demand for wheat in new markets, especially in countries where wheat is not grown. Wheat grain comprises three major groups of components: Starch, proteins, and cell wall polysaccharides, accounting for about 90% of the dry weight [1]. Although proteins are a minority component compared to starch, they are important for the technological properties of wheat. About 80% of the total grain proteins are formed by gluten proteins, which are responsible for the processing properties of wheat flour that enable the manufacture of bread, pasta, noodles, and a wide range of other baked products and ingredients. However, gluten is not a single protein but a mixture, also denoted as prolamins because of the high content of the amino acids proline and glutamine. Classically, gluten proteins are divided into two major fractions, which are different not only in terms of structure and functionality, but also in immunogenicity: (i) The gliadins, which are monomeric, subdivided into ω-, γ-, and α/β-gliadin fractions, and (ii) The glutenins, which form large polymers, comprising of high molecular weight (HMW) and low molecular weight (LMW) subunits. The gliadins contribute extensibility, whilst the glutenins contribute elasticity to the wheat dough. Considering genetic and proteomic data, a typical bread wheat cultivar may contain about 60 different gluten proteins in the grain, of which 30 are gliadins [2]. Gluten is also present in other species of the *Triticeae* tribe, which include barley and rye.

Several pathologies or digestive disorders are related to gluten consumption: Coeliac disease (CD), Non-coeliac gluten sensitivity (NCGS), Wheat allergy, and Wheat-dependent exercise-induced anaphylaxis (WDEIA). CD is a systemic immune-mediated disorder elicited by gluten from wheat, barley, and rye in genetically susceptible individuals. Prevalence of CD varies worldwide but it ranges from 0.3 to 2.4% in western countries [3], where some studies have shown that the incidence of this pathology may have increased over time [4,5]. NCGS is a new digestive disorder with symptoms similar to CD, though its incidence is higher than CD [6]. In NCGS, the autoantibodies that are typical of CD are usually absent; however, an increase in intraepithelial lymphocytes may be found in NCGS, since it is a cause of microscopic enteritis [7]. Gluten from wheat seems to be the primary trigger of NCGS, but other components of the diet, such as metabolic proteins called α-amylase/trypsin inhibitors (ATI) [8] and fermentable oligo-di-mono-saccharides and polyols (FODMAPs), have been identified as important factors for the development of symptoms [9,10]. These findings could indicate that NCGS might not be a separate entity from irritable bowel syndrome (IBS) [11] or they could be distinct entities with some overlapping features [12].

For both CD and NCGS patients, symptoms improve or disappear entirely, when gluten is withdrawn from the diet. Therefore, a gluten free diet (GFD) is at present the most effective treatment available for both patients. However, the nutritional profile of GFDs is of concern, as GFDs are characterized by an unbalanced intake of nutrients, with an excess of saturated and hydrogenated fatty acids; low quantities of alimentary fiber, micronutrients, and vitamins; low contribution to the recommended daily protein intake; and a high carbohydrate content, yielding an increase in the glycemic index [13]. At the same time, a GFD can be detrimental to gut health as it leads to a reduction in the populations of bacteria that are generally regarded as healthy (*Bifidobacterium*, *B. longum*, and *Lactobacillus*), while populations of potentially unhealthy bacteria increase, parallel to reductions in the intake of polysaccharides after following the GFD [14].

The down-regulation of immunodominant gluten peptides by RNA interference (RNAi) in wheat is an excellent approach for reducing gluten toxicity. Protein extracts from flour made from wheat lines where all gliadin fractions have been down-regulated show a reduction in the extracts capacity to stimulate the human leukocyte antigen (HLA)DQ2- and DQ8- restricted T-cell clones in CD patients [15]. Among all the low-gliadin lines generated by RNAi, the E82 line was outstanding as it contained very few CD immunogenic epitopes [16], and bread made using this line showed notable favorable sensory properties and improved nutritional properties [17]. Moreover, the whole-wheat flour of the E82 line administered to rats at three increasing doses for 90 days did not induce any adverse effects, and there was no difference between rats that were fed with the wild type wheat [18].

Our body is colonized by trillions of microorganisms in a symbiotic state, and these populations have fundamental roles in health and disease. Microbial communities in the gut are involved in the well-being of the innate and adaptive immune system. This nexus between nutrient metabolism and the immune system occurs at different levels, from the sensing of nutrients to endocrine signaling. The host microbiome has been associated with vitamin production, lipid metabolism regulation, the regulation of gene expression, and energy extraction from food [19,20,21]. Intestinal microbiota changes may be involved in the development of several diseases; thus, the importance of keeping a balanced gut microbiota [22,23,24]. Other works have suggested that dietary interventions may be useful to restore potentially beneficial gut microbiota groups within the gut [25]. We also know that it is possible to intervene in microbiota in a rapid specific manner with diet changes [26]. Most studies report an imbalance in the gut flora, called dysbiosis, in CD patients treated or untreated with a GFD, and it is likely that this contributes to some persistent gastrointestinal symptoms [27].

Dysbiosis in NCGS causes gut inflammation, diarrhea, constipation, visceral hypersensitivity, abdominal pain, a dysfunctional metabolic state, and issues with peripheral immune and neuro-immune communication [28]. In the context of this study, the link between the intestine and the brain, the known Gut-Brain axis takes on a key role, since several studies postulate that an increase in intestinal permeability could allow gluten peptides to cross the intestinal barrier, enter to the bloodstream and ultimately cross the blood brain barrier causing neuroinflammation among other symptoms [29]. In line with this, it has been reported that changes induced by food and more specifically changes in the intestinal microbiota may promote various pathophysiological mechanisms, neuroinflammation, and cognitive dysfunction, through the Microbiome-Brain-Gut axis [30]. Hence, dysbiosis, gut inflammation, and chronic dyshomeostasis are of great clinical relevance and we should consider them in the diagnosis and treatment of both NCGS and CD.

The aim of the present study was to evaluate the acceptability, digestibility, and safety of a bread made using flour from the E82 line with all gliadins strongly downregulated, in volunteer NCGS patients, in comparison with a GFD. In addition, gut microbiota shifts after the consumption of both GF and E82 breads were also investigated.

## 2. Materials and Methods

### 2.1. Subjects

Eleven patients with non-celiac gluten sensitivity (NCGS) that fulfilled the Salerno criteria [31] and had been on a gluten-free diet for at least the previous 6 months were included in the trial. The age range was 31–57 years, and 9/11 were female. All subjects showed negative serology for celiac disease (tissue-transglutaminase IgA antibodies) and the duodenal biopsy results presented normal duodenal villi architecture. HLA-DQ2+ was present in five of the subjects, whilst the remaining eight showed a different HLA-DQ2 or DQ8 haplotype. Of the 11 subjects initially selected, one withdrew during the first days of the trial for personal reasons (#6, female and HLA-DQ2 negative). The remaining 10 subjects followed the full protocol through the different phases and completed the clinical and sensory questionnaires.

All study participants provided informed consent, and the study design was approved by the local ethics committee of the Hospital of León (approval number 1626).

### 2.2. Study Design

The study was performed in two phases (Basal and E82), each of which had a duration of seven days.

Basal phase: Strict gluten-free diet (GFD) for seven days. During this phase, the subjects consumed the gluten-free bread which they habitually eat at home (100–150 g bread daily). At the end of this phase, Clinical Questionnaires based on Gastrointestinal Symptom Rating Scale (GSRS) and Sensory Questionnaires on palatability and acceptability of the bread were completed, and stool samples (Basal phase samples) were collected.

E82 phase: Continuation of the basal GFD, but with the substitution of the gluten-free bread with the low-gliadin bread E82 for seven days. Patients were instructed to consume a minimum of 100 g and a maximum of 150 g of the E82 bread daily. At the end of this phase, the Clinical and Sensory Questionnaires were completed, as in the Basal phase, and stool samples (E82 phase samples) were collected.

The GSRS questionnaire is a validated, self-administered questionnaire that includes 15 questions, which assess severity of gastrointestinal (GI) symptoms using a 7-point Likert scale in five domains: Indigestion, diarrhea, constipation, abdominal pain, and reflux [32]. The severity of the symptoms reported in the GSRS increases with increasing score.

### 2.3. Wheat Sample Preparation

The low-gliadin wheat line E82 (*Triticum aestivum* cv BW208) was obtained as reported in Reference [15]. The line E82 was authorized to be grown in the field in the village of Fuente Palmera (Córdoba, Spain) under notification number B/ES/13/20 (http://gmoinfo.jrc.ec.europa.eu/gmp_report.aspx?CurNot1/B/ES/13/20), according to the legislation for the release of genetically modified higher plants.

After harvesting, the grains were ground in a Buhler mill (Bühler AG, Gupfenstrasse 5, 9240 Uzwil, Switzerland) and white flour was vacuum stored at 4 °C till the preparation of the breads.

#### Preparation of the Low-Gliadin Breads

The ingredients for the low-gliadin E82 bread provided in the E82 phase were: flour (2.4 kg), water (1.6 L), and salt (12 g). The ingredients were mixed for 10 min and allowed to stand at 15 °C for 14 h and then at 25 °C for 4 h. For bread making, the mixture, yeast (48 g), and salt (36 g) were mixed to form a dough and then it was left to rest for 45 min at room temperature. The fermentation and baking process was carried out as described in Reference [33].

After cooling, the loaves were cut into slices and frozen in portions. The bread, stored at the Hospital of León, was made available to the subjects for the E82 phase. The breads were supplied frozen to the test subjects for defrosting immediately before consumption.

### 2.4. Analysis of the Flour and Bread using RP-HPLC and R5 Competitive ELISA

The gliadin fractions from 100 mg samples of flour and bread were extracted and analyzed by Reverse-phase high-performance liquid chromatography as described in Reference [34]. Briefly, gliadin extracts (40 μL) were applied to a 300SB-C8 reverse phase analytical column (4.6 × 250 mm, 5 μm particle size, 300 Å pore size; Agilent Technologies, Santa Clara, CA, USA) using a 1200 Series Quaternary LC System liquid chromatography (Agilent Technologies, Santa Clara, CA, USA), with a DAD UV-V detector. Absorbance was monitored with the DAD UV-V module at 210 nm.

Flour and bread samples from the low-gliadin wheat were analyzed for gluten content (parts per million) at the Centro Nacional de Biotecnología (CNB-CSIC) using a competitive ELISA, based on the monoclonal antibody R5 as described in Reference [35]. The assay was performed in triplicate.

### 2.5. Collection of Faecal Samples

Subjects collected 2–4 g stool sample in a sealed container after recording food intake at the end of both the basal phase and the E82 phase. Then, the stool samples were delivered within the next two hours after deposition and stored at −80 °C until processing. All samples were identified and labelled with a randomized numeric code.

The MoBioPowerSoil (QIAGEN, Venlo, The Netherlands) kit was used to extract the DNA from faecal samples according to the manufacturer’s instructions, including an initial high shaking step to improve lysis with a TissueLyser II machine (QIAGEN). DNA was quantified using a NanoDrop ND-1000 spectrophotometer (Nanodrop Technologies, Wilmington, DE, USA).

### 2.6. Quantification of the Gluten Immunogenic Peptides (GIP) in Stool Samples

For the study, a total of 20 stool samples were analyzed from the 10 subjects. The extraction and quantification method used was as described in Reference [33]. Each sample was assayed in duplicate and at least two different aliquots from each sample were tested on different days.

### 2.7. Microbiota and Phylogenetic Analysis of the Sequencing Reads

The bacterial 16S rRNA gene V1-V2 hypervariable regions of the 36 samples (nine patients with four samples each: one for each phase of the study and two DNA repetitions for each phase), were amplified in polymerase chain reactions (PCR) using the universal bacterial primers 8F (AGAGTTTGATCMTGGCTCAG) and 357R (CTGCTGCCTYCCGTA), complemented with 8nt index and Illumina adapter sequences. The PCR amplification and single-end sequencing of the V2 hypervariable region was carried out according to Reference [36]. 

The 16S rRNA sequences obtained were analyzed using the Quantitative Insights into Microbial Ecology (QIIME) pipeline (version v1.9.1, http://qiime.org/)) with default parameters [37] as indicated in References [25,36]. Reads were clustered using the de novo Operational Taxonomic Unit (OTU) picking protocol that assigned reads to reference sequences from Greengenes v13-8 and SILVA v128 [38], and taxonomy assignation was assigned using the Greengenes v13-8 and SILVA V128 pre-clustered at 97% identity. Rarefaction curves of alpha-diversity indexes, including estimates of community richness (Chao1 estimator, Good’s coverage, the observed number of OTUs present in each sample, and Phylogenetic diversity (PD)) and beta diversity were estimated using weighted and unweighted UniFrac distances as in Reference [39]. Prior to that, with the purpose to retain all samples, each library was sub sampled to an even sequencing depth of exactly 10,696 sequences per sample.

### 2.8. Statistical Analysis

Data were analyzed with the statistical software Statistix version 10 (Analytical Software, Tallahassee, FL, USA). A paired t-Test was used to compare symptoms and global acceptance.

Differences in alpha diversity between both phases were estimated using a non-parametric test and Monte Carlo permutations, for each of the three alpha diversity estimators (PD whole tree, chao1, and observed OTUs). We also estimated the existence of statistically significant differences between groups for the beta diversity unweighted and weighted UNIFRAC distances, using a PERMANOVA test.

To assess whether specific differences occurred in some bacterial taxa between phases, we selected the taxa that were present in at least 80% of the samples per each phase. The non-parametric Kruskal-Wallis test was used to compare the existence of differences in the relative abundance of the bacterial taxa between phases using the PASW statistical software, version 20.0 (IBM Inc., Chicago, IL, USA).

## 3. Results

### 3.1. Flour and Bread Characteristics, Faecal GIP Content, Clinical Symptoms, and Acceptability

**Figure 1:**
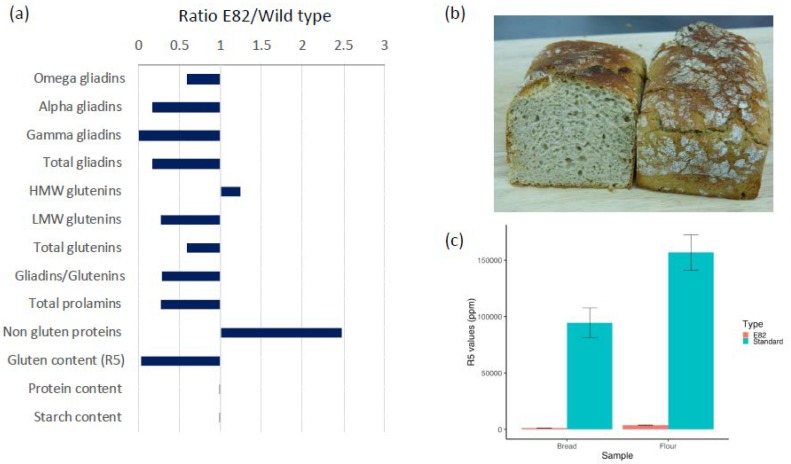
Low-gliadin E82 bread characteristics: (**a**) E82/wild type ratio for main grain components; (**b**) low-gliadin breads used for the dietary intervention, and (**c**) gluten content values (ppm) as determined using the monoclonal antibody R5 for E82 and standard flours and breads.

Figure 1, panel (a) shows the fold changes for the main flour components of the E82 line in relation to the wild type. The analysis of the low gliadin line E82 using RP-HPLC showed a very low content of gliadins, with a total reduction of nearly 90%, in comparison to its wild type (Figure 1a). The RNAi silencing was very effective, and all three gliadin groups were strongly down regulated in this line, particularly the gamma and alpha-gliadins. As reported previously in Reference [15], the low molecular weight (LMW) glutenins were also affected by the down regulation in this line, while the high molecular weight (HMW) glutenins were increased (Figure 1a). Overall, a reduction of about 60% in the total prolamins was recorded, which was apparently compensated by a significant increase in the non-gluten proteins, leading to a total protein content similar to the wild type. Determination of gluten content by the R5 antibody-based ELISA showed a reduction of nearly 97% for the E82 line in comparison to that of the wild type (Figure 1a).

Bread derived from the low-gliadin line E82 was made using a simple formulation with no additives and long fermentation times. The final bread product (Figure 1b) was provided frozen to subjects with specific instructions for defrosting before consumption. The analysis of gluten content (ppm) in the flour and bread by competitive ELISA using the R5 monoclonal antibody showed a significant reduction in the levels of gluten after the fermentation and baking of flour to bread. The content of gluten in flour was 3545 ppm (mean value), while in bread it was 1130 ppm (mean value), which was more than three times less (Figure 1c). The gluten content (ppm) of standard flour and bread, made in the same bakery, were 166,400 and 93,966 ppm, respectively [33], which were much higher than that of the E82 line reported here.

**Figure 2:**
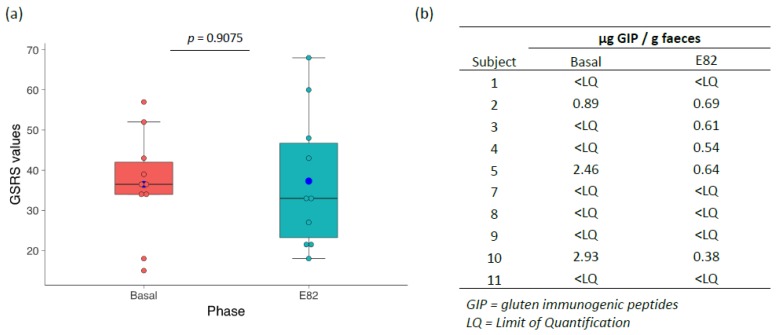
(**a**) Clinical symptoms evaluated at each of the phases of the study by the Gastrointestinal Symptom Rating Scale (GSRS) questionnaire; (**b**) Comparison of the faecal Gluten Immunogenic Peptides (GIP) content after consumption of gluten-free bread (Basal) and the low-gliadin bread (E82).

Clinical symptoms were evaluated at each phase of the study by the GSRS questionnaire. No significant differences in the overall score were observed between the two phases (Figure 2a). For the basal phase, a mean score of 36.5 was obtained, and for the E82 phase, the mean score was 37.3 (*p* = 0.9075). Furthermore, the five domains analyzed by the GSRS (indigestion, diarrhea, constipation, abdominal pain, and reflux) showed similar scores after consuming the low-gliadin E82 bread compared with the basal GF bread. Moreover, the patients scored both diets similarly, except patient #1, whose gave a higher score to the E82 bread, and patient #11, who scored the GF bread higher (Figure S1).

All subjects followed a GFD, and after completing each of the two phases (Basal and E82 phases), the presence of GIP was studied in the faecal samples. During the basal phase, three patients showed values for GIPs that were higher than the limit of quantification, with a maximum of 2.93 μg/g feces. For the E82 phase, all patients showed GIP levels below 0.69 μg/g feces, with five patients showing an undetectable GIPs content (Figure 2b). The results obtained in this last phase contrasted with those previously obtained for subjects who included standard breads containing gluten in their diet (GIP excretion between 0.30 and 75.68 μg/g feces) [33].

**Figure 3:**
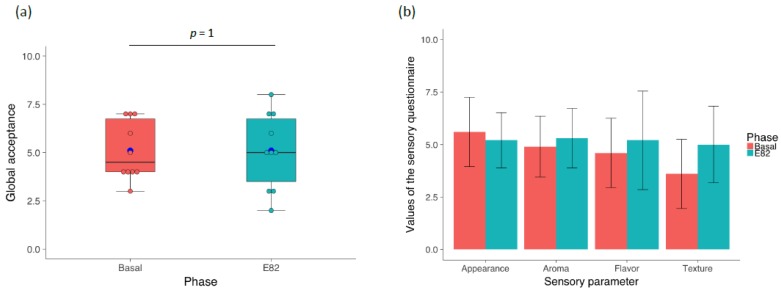
(**a**) Differences in global acceptance of the two phases of the dietary intervention; (**b**) Values of the sensory questionnaire for appearance, aroma, flavor, and texture for the gluten-free bread (Basal) and the low-gliadin bread (E82).

The Sensory Questionnaires evaluated five different aspects of the breads consumed in each of the basal and E82 phases, on a ten-point scale: appearance, aroma, flavor, texture, and overall acceptability. The results of the overall acceptability scores are presented in Figure 3, panel (a). For the basal diet, values ranged between 3 and 7 (mean 5.1), whilst for the E82 phase, the values were between 2 and 8 (mean 5.1). There was no significant difference in the global acceptance between both types of breads (*p* = 1). All aspects, except for appearance, in the Sensory Questionnaires were scored higher for the E82 bread, though with no significant difference with the basal phase (Figure 3b).

### 3.2. Diversity, Relative Abundance, and Changes of the Gut Microbiota

In total, 8 phyla, 16 classes, 20 orders, 33 families, 46 genera, and 22 species, were identified in all the samples. On average, the phyla with the highest abundance were *Firmicutes* (94.9%), *Actinobacteria* (3.5%), *Proteobacteria* (0.8%), and *Bacteroidetes* (0.2%). At the family level, the most abundant were *Ruminococcaceae* (30.9%), *Lachnospiraceae* (23.6%) *Erysipelotrichaceae* (12.3%), *Veillonellaceae* (10.4%), *Clostridiaceae* (3.8%), *Streptococcaceae* (2.8%), and *Coriobacteriaceae* (2.4%). At the genera level, the most abundant were *Dialister* (4.7%), *Catenibacterium* (4.61%), *Ruminococcus* (3.1%), and *Faecalibacterium* (3.0%). Finally, at the species level, the most abundant were *Faecalibacterium prausnitzii* (3.0%), *Eubacterium biforme* (1.1%), and *Collinsella aerofaciens* (0.9%). Other taxa that comprise the gut microbiota, for example, the phyla *Fusobacteria*, *Verrucomicrobia,* and *Cyanobacteria*, were also detected but the relative abundance of the taxa was negligible (less than 0.1%).

Satisfactory Good’s coverage of the diversity was obtained for all samples, since the mean value was 92.6. We did not find any significant differences in bacterial diversity between the basal phase and the E82 phase, with any of the alpha diversity estimators used, PD whole tree (*p* = 0.357), chao1 (*p* = 0.232), and observed OTUS or Richness (*p* = 0.265) (Figure S2). Similarly, the Principal Coordinate Analysis (PCoA) on the unweighted and weighted UniFrac distances did not show any significant differences in the beta diversity between both phases (*p* = 0.165 and *p* = 0.059, respectively) (Figure S3).

At the phylum level, we observed a lower abundance of the *Actinobacteria* phylum in the E82 phase compared to the basal phase (*p* = 0.031) but observed no differences between the phases for any other phylum (Figure S4).

At the class and order levels, we observed a significantly lower abundance in the E82 phase compared with the basal phase for members of the *Coriobacteriia* class (*p* < 0.01), as well as the *Bacteroidales* and the *Coriobacteriales* orders (*p* < 0.01 and *p* = 0.033, respectively) (Figure S4).

**Figure 4:**
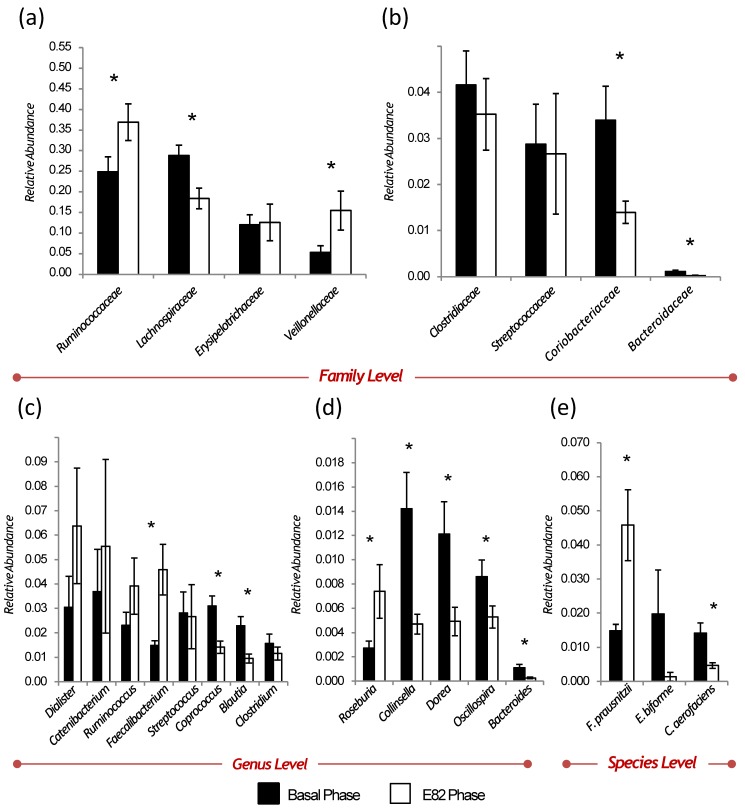
Differences in the gut microbiota composition between the basal phase and the E82 phase at family, genus, and species level. Values are the mean ± S.E.M. The statistically significant differences between each phase were tested using the non-parametric Kruskal-Wallis test. * *p* < 0.05. In the bar diagrams are represented: (**a**) bacterial families whose average relative abundance is greater than 10%; (**b**) bacterial families whose average relative abundance is less than 10% but greater than 1%, with the exception of the family *Bacteroidaceae* (0.07% of average relative abundance); (**c**) bacterial genera whose average relative abundance is greater than 1%; (**d**) bacterial genera whose average relative abundance is less than 1%; (**e**) bacterial species whose average relative abundance is ≥1%.

At the family level, we found a significantly higher abundance of *Veillonellaceae* (*p* = 0.036) and *Ruminococcaceae* (*p* = 0.035) in the E82 phase (Figure 4). In contrast, the *Lachnospiraceae*, *Bacteroidaceae*, and *Coriobacteriaceae* families showed a significantly lower abundance in the E82 phase compared to the basal phase (*p* < 0.01, *p* < 0.01 and *p* = 0.03, respectively) (Figure 4).

Otherwise, we also observed significant differences between the phases for several bacterial genera. Therefore, a lower abundance in the E82 phase for *Oscillospira* (*p* = 0.038), Dorea, *Blautia*, *Bacteroides*, *Coprococcus* (all *p* < 0.01), and *Collinsella* (*p* = 0.013) genera was observed. Conversely, we observed a higher abundance in the E82 phase in the *Roseburia* and *Faecalibacterium* genera compared to the basal phase (*p* < 0.01 and *p* = 0.022, respectively) (Figure 4).

Finally, at the species level, we observed a lower abundance in *Collinsella aerofaciens* (*p* = 0.013) and a higher abundance in *Faecalibacterium prausnitzii* (*p* = 0.022) in the E82 phase compared to the basal phase (Figure 4).

## 4. Discussion

Our study shows that the consumption of low-gliadin E82 bread for one week had great acceptability by the NCGS patients, since clinical symptoms were the same as those seen with the gluten-free bread, and no differences in the sensory parameters were reported. In addition, we observed potentially positive changes in the composition of the subjects’ gut microbiota after the E82 bread was consumed. The principal findings were an increase in the relative abundance of the *Roseburia* and *Faecalibacterium* genera, and a decrease in the *Bacteroides*, *Blautia*, *Dorea*, *Coprococcus*, and *Collinsella* genera after the E82 phase, which matched a microbial profile that was previously shown to have a key role in the improvement of gut permeability and anti-inflammatory properties.

NCGS is an intestinal disorder, with increasing prevalence especially in western populations, which has led to the increased demand for food products without or with low levels of gluten [9]. The E82 bread is distinguished by having a very low gliadin content and reduced LMW glutenins, but it has a high non-gluten protein content, which leads to a protein content that is comparable to that of the wild type. Moreover, the low gliadin bread has increased lysine content, with increments that ranged between 24–67% compared to the wild type [17]. This bread has shown a significant decrease in gluten levels after the fermentation and baking of the flour to bread. Long fermentation processes and baking could be implicated in this significant reduction of the gluten from flour to bread. From the analysis of gluten proteins in the E82 and wild type, the most important aspect observed was the reduction in the levels of γ-gliadins and α-gliadins, as well as in the ratio of gliadins to glutenins. These results explained the previous findings that protein extracts from the E82 flour were unable to elicit responses in T-cell lines reactive with omega gliadins and caused highly reduced responses with T-cell lines reactive to alpha and gamma epitopes [15].

Therefore, the E82 bread may be of interest to those who want to reduce gluten intake and it is a possible alternative to GF-bread for use in GFD diets. The GFD is the only effective treatment for celiac disease and it is also beneficial for NCGS. Nevertheless, a GFD is very complicated to follow, socially inconvenient, and expensive. Both the high processing of gluten free foods and the number of additives used to mimic the functionality of gluten are related with the problem of poor acceptability in the population [40,41,42].

In this study, responses to the Sensory Questionnaire showed that the overall acceptance was the same when comparing the E82 bread to the gluten free bread of choice consumed by the NCGS volunteers. None of the sensory parameters evaluated showed significant differences when the two types of breads were compared, but aspects such as texture, aroma, and flavor showed a slight tendency to increase with the consumption of the E82 bread. Moreover, the GSRS Questionnaire that assesses the severity of five GI-symptoms (indigestion, diarrhea, constipation, abdominal pain, and reflux) showed that the NCGS patients’ scores were similar between both phases. Only patient #1 gave a higher score to the E82 bread (Figure S1). Three and five patients showed levels of GIPs that were higher than the limit of quantification after consumption of the GF and E82 breads, respectively. In the case of the GF bread, this could indicate the ingestion (voluntary or not) of food containing gluten during that phase. However, transgressions during the E82 phase cannot rule out either. Additionally, the levels of GIP excreted during the E82 diet ranged between 0 and 0.61 μg/g, whilst the range of GIP excretion values in the normal diet were 0.30 and 75.68 μg/g sample [33], showing a significant decrease in GIP excretion with the E82 diet. Taken together, these results showed a great acceptability of the E82 bread by NCGS patients, which further supported the idea that the E82 bread could be a positive choice as part of a GFD for these patients.

Previous in vitro studies [43,44], described how the Gluten Friendly Bread (GFB) produced a beneficial modulation of the gut microbiota in both healthy and CD subjects, increasing the bacterial populations of the genera *Bifidobacterium* and *Lactobacillus*, as well as an increase in the production of short chain fatty acids (SCFAs). The first study was based on a batch-culture fermentation system, which simulated the distal region of the human large intestine, whilst the second study used a continuous colonic model in three stages imitating the proximal, transverse, and distal human colon. Although both studies opened a new horizon to explore using gut simulating systems, they presented some limitations such as the number of samples and that these were in vitro studies, making it essential to validate the results in human intervention studies in vivo.

Our present study evaluated in vivo the effect of bread E82, which could be considered a GFB, on the composition of the intestinal microbiota in eleven patients with NCGS. Our results based on the intestinal microbiota composition showed that the E82 bread consumption induced an increase in the relative abundance of the genus *Faecalibacterium*, specifically of the species *F. prausnitzii*, and a decrease in the relative abundance of other genera, such as *Bacteroides*, *Blautia*, *Dorea*, *Coprococcus*, and *Collinsella* (specifically the species *C. aerofaciens*). This microbial profile has been previously described in several studies [45,46,47] to have a positive impact on gut permeability, and therefore, a better functioning of the intestinal barrier. Although there are several studies with controversial results regarding intestinal permeability and NCGS and EC [48,49,50,51,52,53,54,55,56], our results could open a future new line of research that studies the contribution of the composition of the intestinal microbiota to the intestinal permeability in these patients.

Another interesting finding in this study was the increase of the most efficient butyrate-producing bacteria, such as the *Roseburia* and *Faecalibacterium* genera (*F. prausnitzii*) [57,58]. Butyrate is a short chain fatty acid produced by intestinal microbial fermentation of undigested dietary carbohydrates in the colon and it modifies the expression of the tight junction proteins in favor of gut barrier preservation and reduced gut permeability [59,60]. These butyrate-producing genera could play an important role in the maintenance of gut health, considering that they are also known for having an anti-inflammatory capacity [61,62].

Our results suggest that the E82 bread consumption favored a less inflammatory phenotype that could be related to the increase of *Faecalibacterium*, an anti-inflammatory genus, and the decrease of *Bacteroides* a proinflammatory genus, thereby improving the gut inflammation characteristics of NCGS patients [47,63]. The change in the relative abundance of these two genera and the association of the genera with the subjects’ inflammatory status was in agreement with the results obtained in this study, related to the quantification of GIPs in stool samples after the E82 phase. It is known that both gluten and GIPs stimulate innate immune responses, which favors the inflammation of the intestinal mucosa in NCGS and CD patients [9]. The measurement of GIPs in stool samples from the volunteers did not show any difference between the basal and E82 phases. These results suggested that the E82 bread could have a similar immunogenic profile as gluten free bread and would promote a healthy inflammation status by its positive effect on gut microbiota composition.

Our study may have the limitation that the intestinal fungal microbiome, also known as the mycobiome, has not been addressed, as well as the relationship between the bacterial and fungal species of the gut microbiota in these patients. Currently, the study of the mycobiome has been limited, but recently it has been demonstrated that it is a key player in the state of health and some human diseases, especially modulating the immune system and producing secondary metabolites in synergy with the intestinal bacteria [64,65]. Therefore, a dysbiosis of the intestinal microbiota (bacterial and fungal microbiota) will cause the dysfunction of the intestinal tissue, ultimately triggering disease susceptibility [65]. Recently, a study investigated the intestinal mycobiome in the Human Microbiome Project (HMP) cohort and it described that the fungal diversity was relatively low in the stool of the healthy subjects [66]. In short, further studies are required to fully understand the potential gut microbiota modification (bacterial and fungal microbiota) that may occur from the consumption of the E82 bread in the prevention of NCGS, as well as other disorders related to gluten.

## 5. Conclusions

In conclusion, our results suggest that the consumption of the E82 bread could be a positive component of the GFD for NCGS patients, since it produces no adverse clinical symptoms, has acceptable sensory parameters, and it induces positive changes in the gut microbiota composition, increasing the butyrate-producing bacteria and favoring a bacterial profile with a key role in the maintenance or improvement of the gut permeability in NCGS patients.

## Supplementary Materials

The following are available online at http://www.mdpi.com/2072-6643/10/12/1964/s1, Figure S1: GSRS values per patient for the two different breads, Figure S2: Alpha diversity estimators. Graphs represented show the rarefaction curves between basal phase and E82 phase that were estimated using a non-parametric test and Monte Carlo permutations at a depth of 10696 sequences per sample for each of the three alpha diversity estimators: (a) PD whole tree; (b) chao1; (c) observed OTUs, Figure S3: Beta-diversity estimators. Graph represented show Principal Coordinate Analysis (PCoA) between basal phase and E82 phase that were estimated using a PERMANOVA test at a depth of 10696 sequences per sample for each beta diversity estimators: (a) unweighted UniFrac distances; (b) weighted UniFrac distances, Figure S4: Differences in the gut microbiota composition between basal phase and E82 phase at phylum, class and order level. Values are the mean ± S.E.M. The statistically significant differences between each phase were tested by non-parametric Kruskal-Wallis test. * *p* < 0.05. (a) Phylum level; (b) Class level; (c) Order level.
