# Supplementary material for: The Dietary Intervention of Transgenic Low-Gliadin Wheat Bread in Patients with Non-Celiac Gluten Sensitivity (NCGS) Showed No Differences with Gluten Free Diet (GFD) but Provides Better Gut Microbiota Profile
Source: Nutrients. 2018 Dec 12;10(12):1964. doi: 10.3390/nu10121964 (PMC6316513; doi:10.3390/nu10121964)
Supplement: Supplementary file 1 [file nutrients-10-01964-s001.zip › Supplementary Files/Figure S2.pdf]

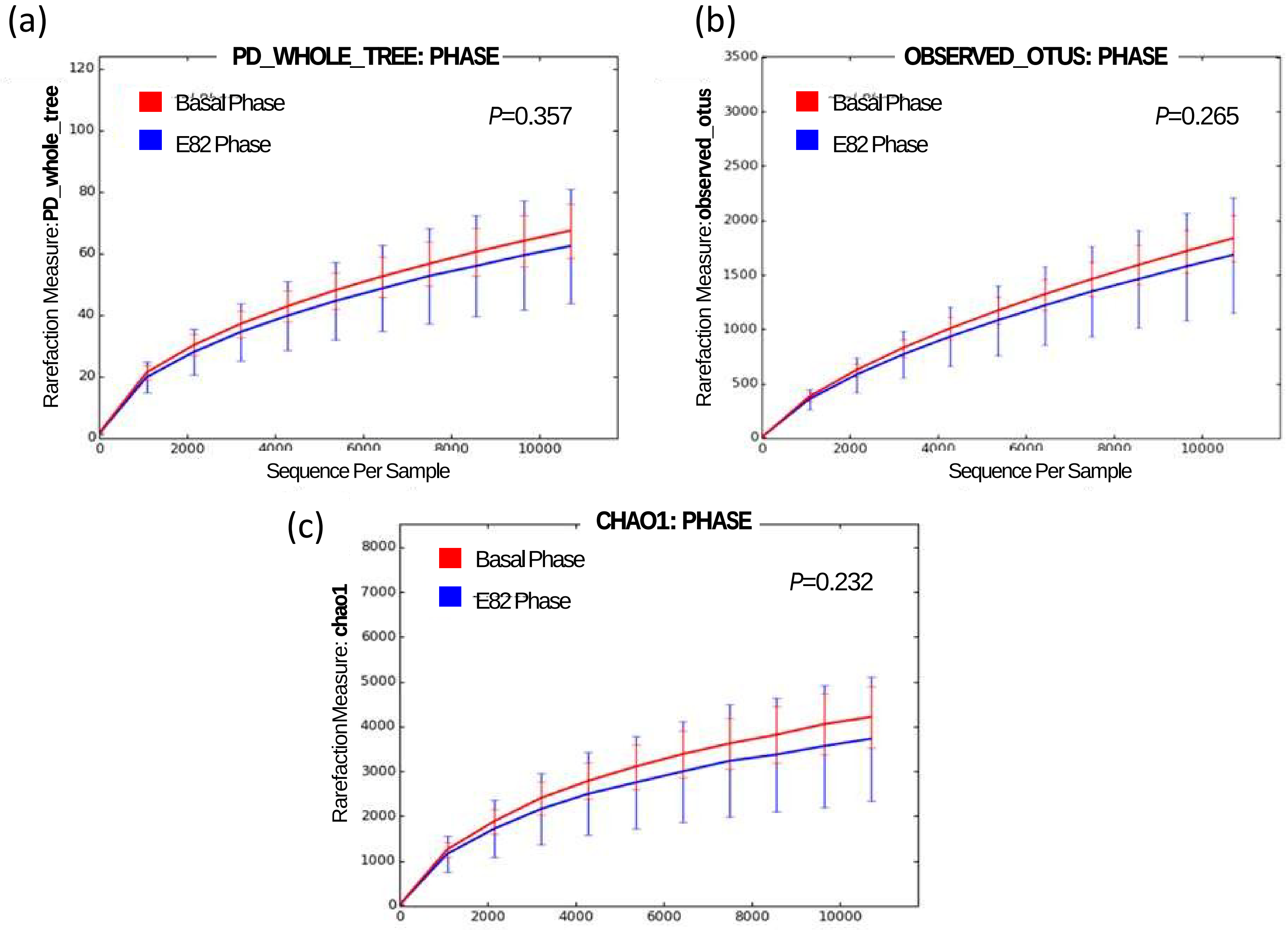

Figure S2. Alpha diversity estimators. Graphs represented show the rarefaction curves between basal phase and E82 phase that were estimated using a non-parametric test and Monte Carlo permutations at a depth of 10696 sequences per sample for each of the three alpha diversity estimators : **(a)** PD whole tree; **(b)** chao1; **(c)** observed otus.
