# Supplementary material for: The Dietary Intervention of Transgenic Low-Gliadin Wheat Bread in Patients with Non-Celiac Gluten Sensitivity (NCGS) Showed No Differences with Gluten Free Diet (GFD) but Provides Better Gut Microbiota Profile
Source: Nutrients. 2018 Dec 12;10(12):1964. doi: 10.3390/nu10121964 (PMC6316513; doi:10.3390/nu10121964)
Supplement: Supplementary file 1 [file nutrients-10-01964-s001.zip › Supplementary Files/Figure S2.pptx]

## Slide 1
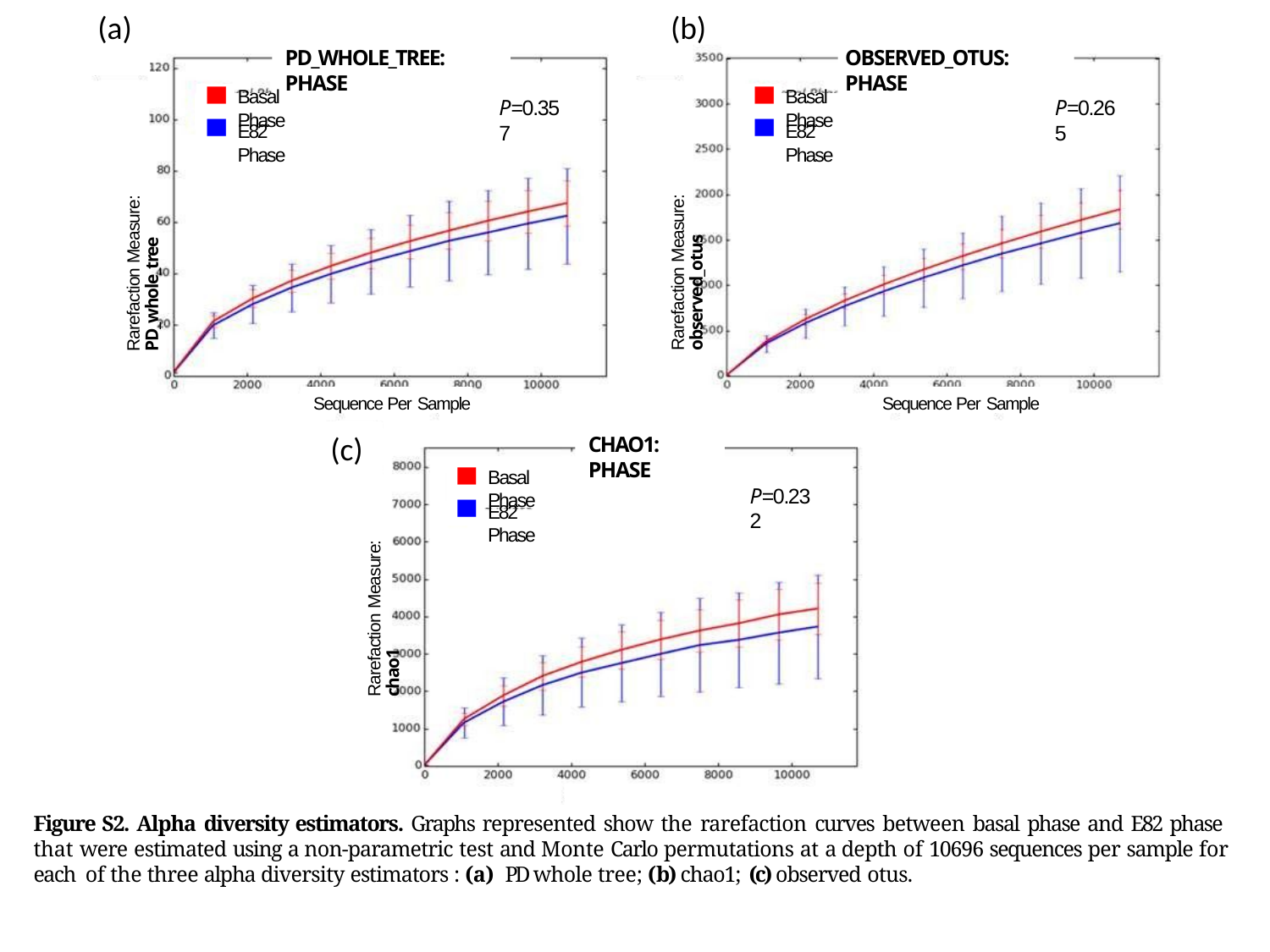

(a)
(b)
PD_WHOLE_TREE: PHASE
OBSERVED_OTUS: PHASE
Rarefaction Measure: PD_whole_tree
Rarefaction Measure: observed_otus
Basal Phase
Basal Phase
P=0.357
P=0.265
E82 Phase
E82 Phase
Sequence Per Sample
Sequence Per Sample
(c)
CHAO1: PHASE
Basal Phase
P=0.232
Rarefaction Measure: chao1
E82 Phase
Figure S2. Alpha diversity estimators. Graphs represented show the rarefaction curves between basal phase and E82 phase that were estimated using a non-parametric test and Monte Carlo permutations at a depth of 10696 sequences per sample for each of the three alpha diversity estimators : (a) PD whole tree; (b) chao1; (c) observed otus.
