# Supplementary material for: The Dietary Intervention of Transgenic Low-Gliadin Wheat Bread in Patients with Non-Celiac Gluten Sensitivity (NCGS) Showed No Differences with Gluten Free Diet (GFD) but Provides Better Gut Microbiota Profile
Source: Nutrients. 2018 Dec 12;10(12):1964. doi: 10.3390/nu10121964 (PMC6316513; doi:10.3390/nu10121964)
Supplement: Supplementary file 1 [file nutrients-10-01964-s001.zip › Supplementary Files/Figure S3.pdf]

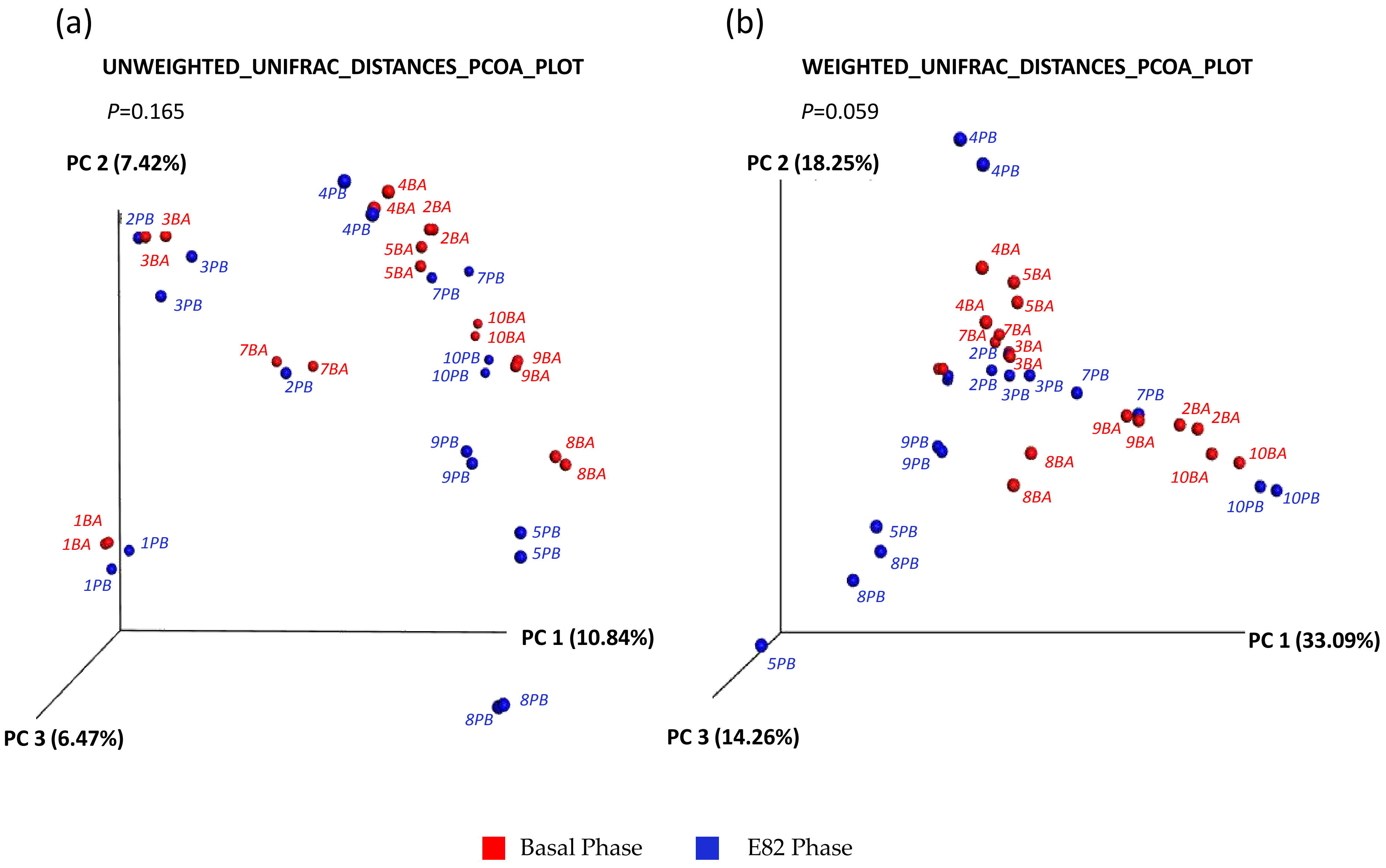

Figure S3. Beta-diversity estimators. Graph represented show Principal Coordinate Analysis (PCoA) between basal phase and E82 phase that were estimated using a PERMANOVA test at a depth of 10696 sequences per sample for each beta diversity estimators: **(a)** unweighted UniFrac distances; **(b)** weighted UniFrac distances.
