# Supplementary material for: The Dietary Intervention of Transgenic Low-Gliadin Wheat Bread in Patients with Non-Celiac Gluten Sensitivity (NCGS) Showed No Differences with Gluten Free Diet (GFD) but Provides Better Gut Microbiota Profile
Source: Nutrients. 2018 Dec 12;10(12):1964. doi: 10.3390/nu10121964 (PMC6316513; doi:10.3390/nu10121964)
Supplement: Supplementary file 1 [file nutrients-10-01964-s001.zip › Supplementary Files/Figure S3.pptx]

## Slide 1
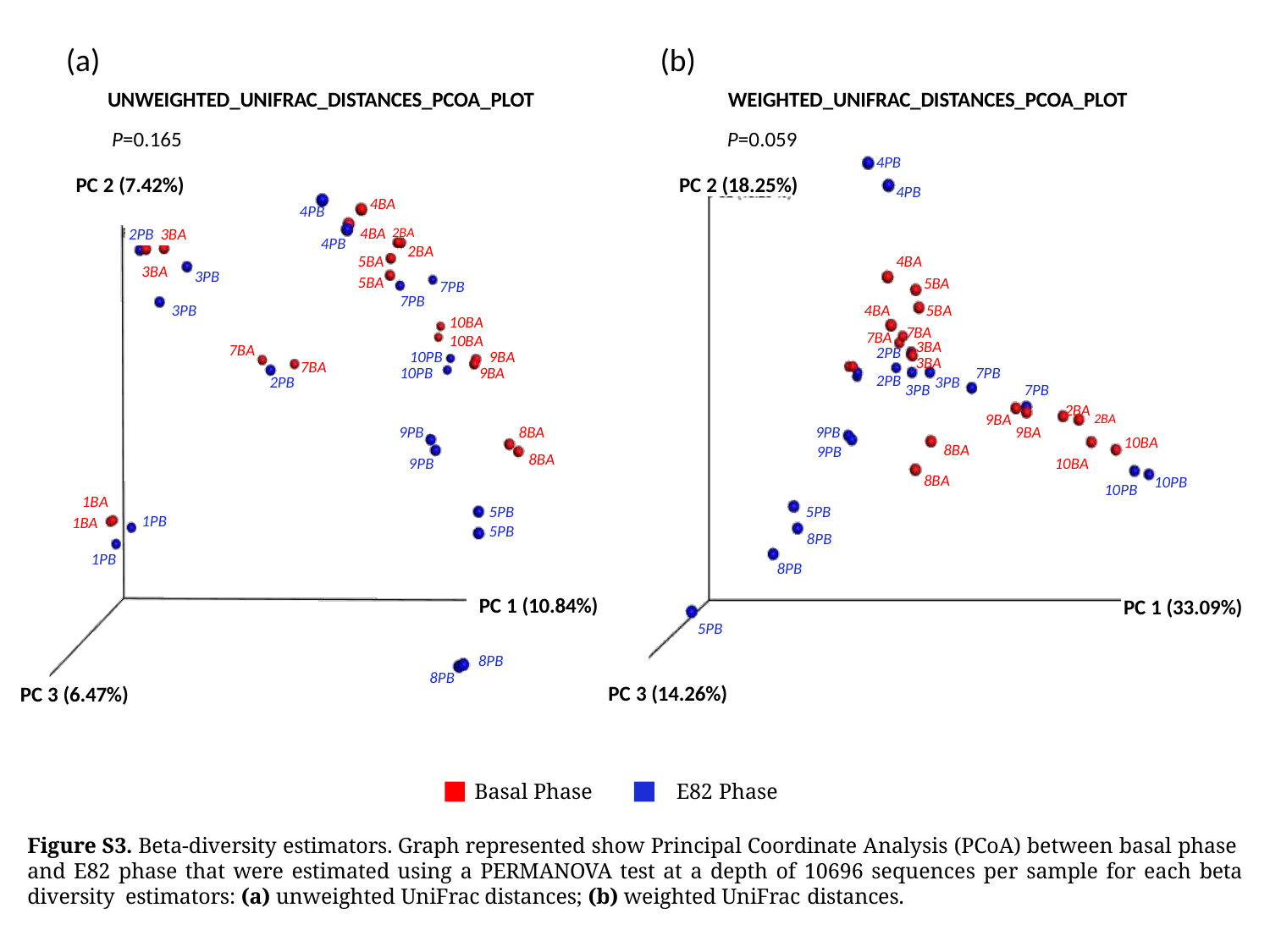

(a)
(b)
UNWEIGHTED_UNIFRAC_DISTANCES_PCOA_PLOT
WEIGHTED_UNIFRAC_DISTANCES_PCOA_PLOT
P=0.165
P=0.059
4PB
4PB
PC 2 (18.25%)
PC 2 (7.42%)
4BA
4BA 2BA
4PB
2PB
3BA
4PB
2BA
5BA
5BA
4BA
3BA
5BA
5BA
7BA
3BA
3BA
3PB
7PB
7PB
3PB
4BA
10BA
10BA
7BA
2PB
2PB
7BA
10PB
10PB
9BA
9BA
7BA
2PB
7PB
3PB
3PB
7PB
2BA 2BA
9BA
8BA
8BA
9PB
9PB
9PB
9BA
10BA
8BA
9PB
10BA
8BA
10PB
10PB
1BA
1BA
5PB
8PB
5PB
5PB
1PB
1PB
8PB
PC 1 (10.84%)
PC 1 (33.09%)
5PB
8PB
8PB
PC 3 (14.26%)
PC 3 (6.47%)
Basal Phase
E82 Phase
Figure S3. Beta-diversity estimators. Graph represented show Principal Coordinate Analysis (PCoA) between basal phase and E82 phase that were estimated using a PERMANOVA test at a depth of 10696 sequences per sample for each beta diversity estimators: (a) unweighted UniFrac distances; (b) weighted UniFrac distances.
