# Supplementary material for: The Dietary Intervention of Transgenic Low-Gliadin Wheat Bread in Patients with Non-Celiac Gluten Sensitivity (NCGS) Showed No Differences with Gluten Free Diet (GFD) but Provides Better Gut Microbiota Profile
Source: Nutrients. 2018 Dec 12;10(12):1964. doi: 10.3390/nu10121964 (PMC6316513; doi:10.3390/nu10121964)
Supplement: Supplementary file 1 [file nutrients-10-01964-s001.zip › Supplementary Files/Figure S4.pdf]

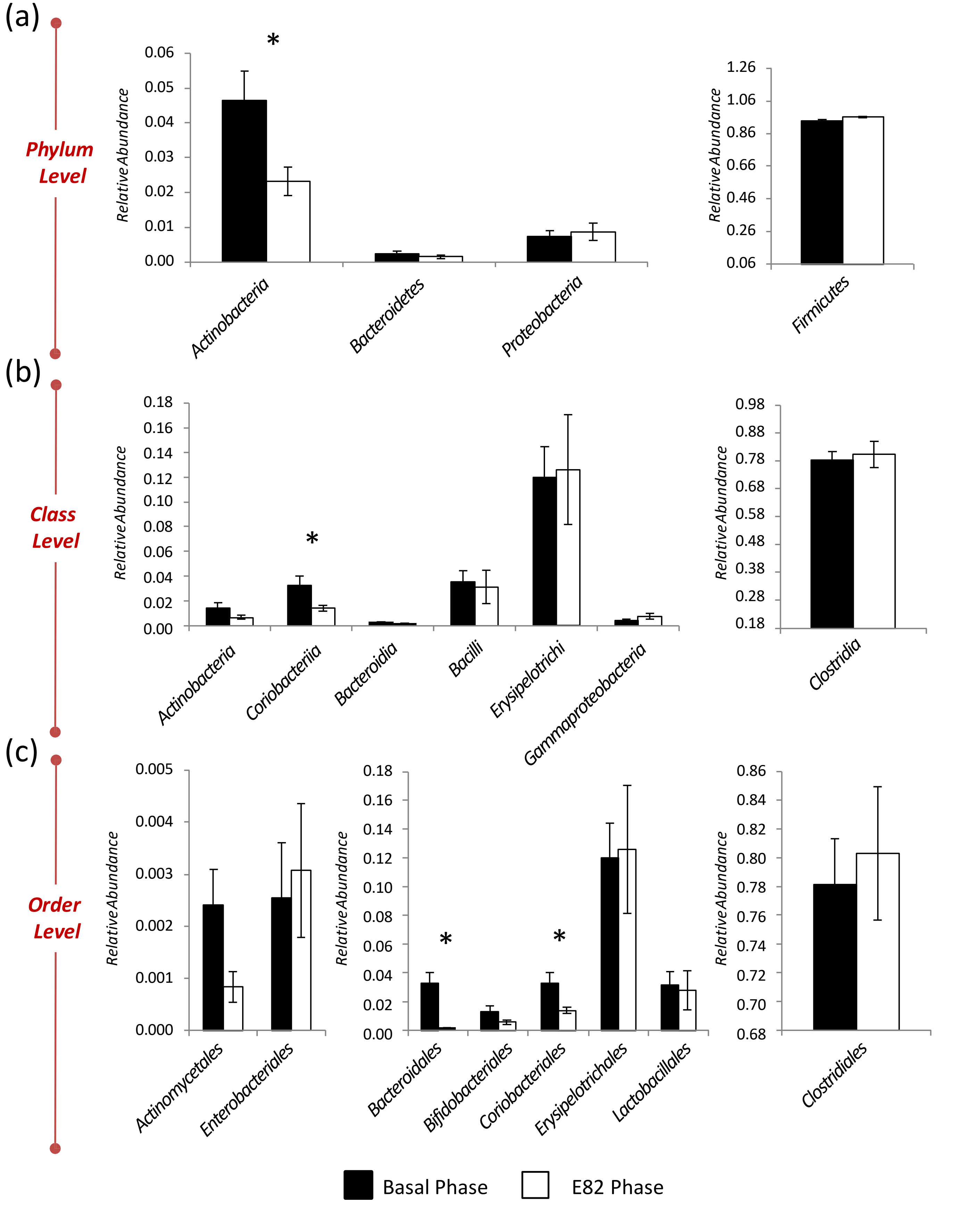

Figure S4. Differences in the gut microbiota composition between basal phase and E82 phase at phylum, class and order level. Values are the mean ± S.E.M. The statistically significant differences between each phase were tested by non-parametric Kruskal-Wallis test. * P<0.05. **(a)** Phylum level; **(b)** Class level; **(c)** Order level.
