# Supplementary material for: The Dietary Intervention of Transgenic Low-Gliadin Wheat Bread in Patients with Non-Celiac Gluten Sensitivity (NCGS) Showed No Differences with Gluten Free Diet (GFD) but Provides Better Gut Microbiota Profile
Source: Nutrients. 2018 Dec 12;10(12):1964. doi: 10.3390/nu10121964 (PMC6316513; doi:10.3390/nu10121964)
Supplement: Supplementary file 1 [file nutrients-10-01964-s001.zip › Supplementary Files/Figure S4.pptx]

## Slide 1
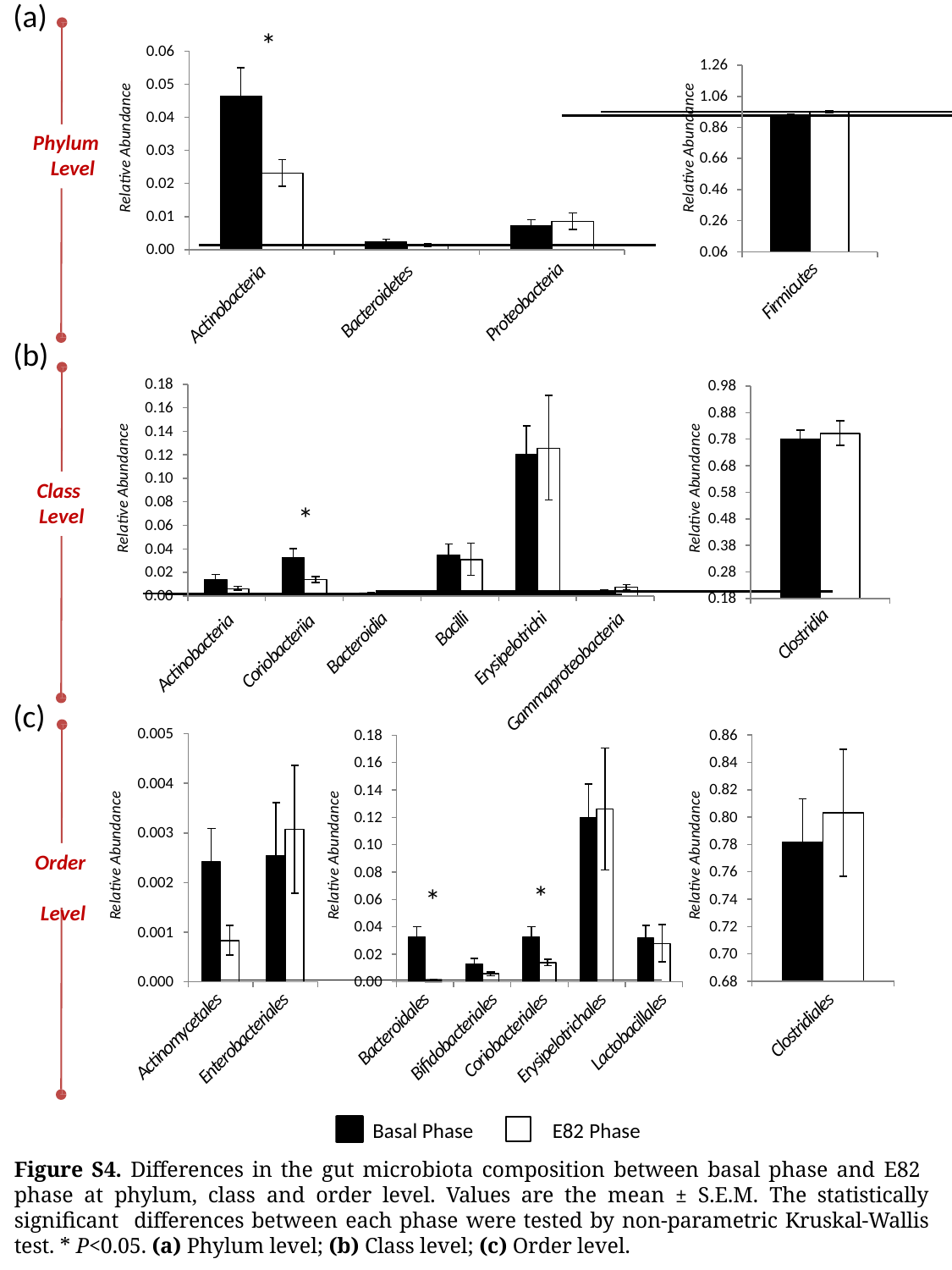

(a)
*
0.06
1.26
0.05
Relative Abundance
Relative Abundance
1.06
0.04
0.86
Phylum Level
0.03
0.66
0.02
0.46
0.01
0.26
0.00
0.06
Firmicutes
Bacteroidetes
Proteobacteria
Actinobacteria
(b)
0.98
0.88
0.78
0.68
0.58
0.48
0.38
0.28
0.18
0.18
0.16
0.14
0.12
Relative Abundance
Relative Abundance
0.10
0.08
0.06
0.04
0.02
0.00
Class Level
*
Bacilli
Clostridia
Bacteroidia
Erysipelotrichi
Coriobacteriia
Actinobacteria
Gammaproteobacteria
(c)
0.86
0.84
0.82
0.80
0.78
0.76
0.74
0.72
0.70
0.68
0.18
0.16
0.14
0.12
0.10
0.08
0.06
0.04
0.02
0.00
0.005
0.004
Relative Abundance
Relative Abundance
Relative Abundance
0.003
Order Level
0.002
*
*
0.001
0.000
Clostridiales
Bacteroidales
Lactobacillales
Coriobacteriales
Actinomycetales
Bifidobacteriales
Erysipelotrichales
Enterobacteriales
Basal Phase
E82 Phase
Figure S4. Differences in the gut microbiota composition between basal phase and E82 phase at phylum, class and order level. Values are the mean ± S.E.M. The statistically signiﬁcant differences between each phase were tested by non-parametric Kruskal-Wallis test. * P<0.05. (a) Phylum level; (b) Class level; (c) Order level.
