# Supplementary figures and images for: The Dietary Intervention of Transgenic Low-Gliadin Wheat Bread in Patients with Non-Celiac Gluten Sensitivity (NCGS) Showed No Differences with Gluten Free Diet (GFD) but Provides Better Gut Microbiota Profile
Source: Nutrients. 2018 Dec 12;10(12):1964. doi: 10.3390/nu10121964 (PMC6316513; doi:10.3390/nu10121964)

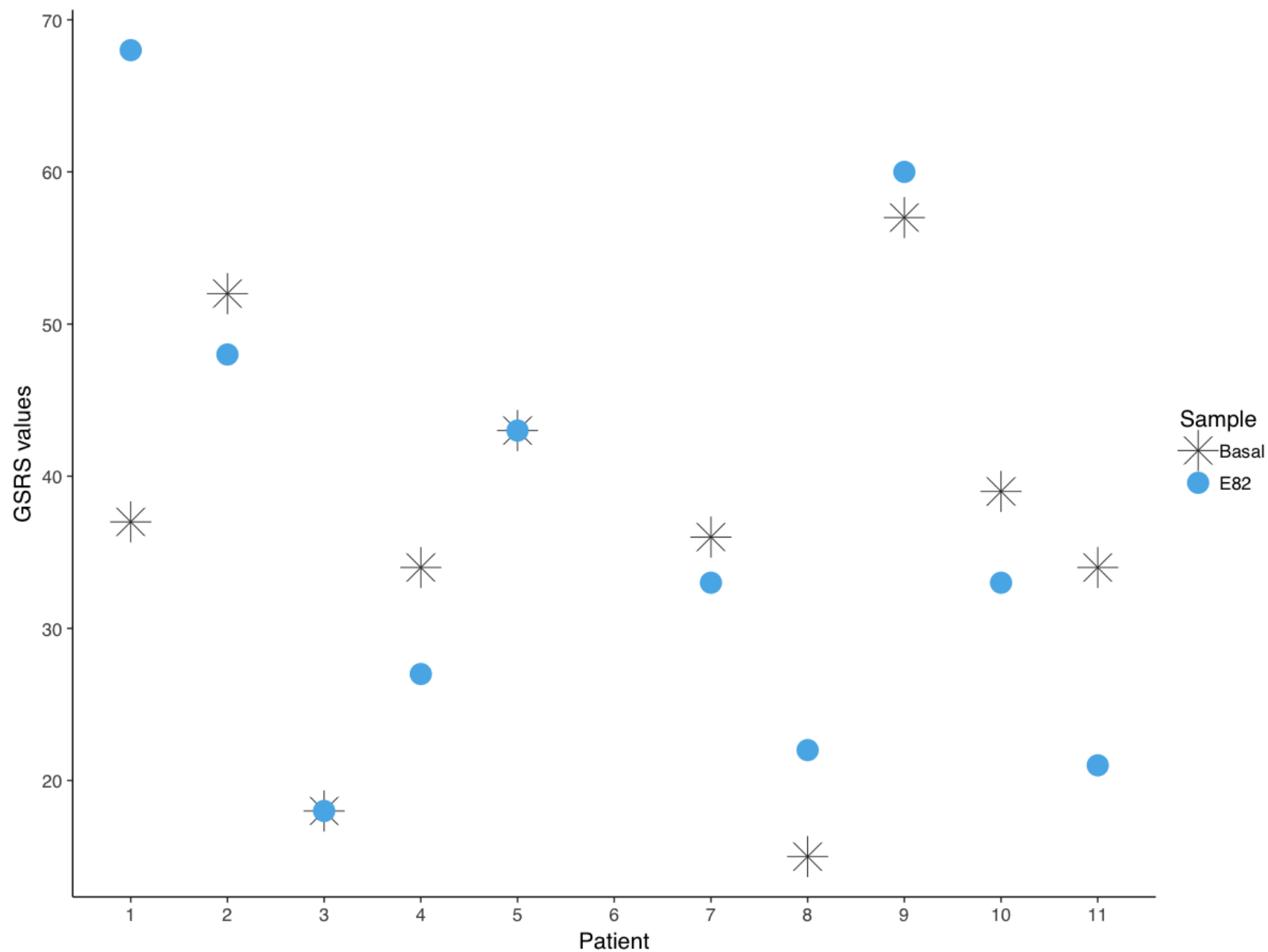

**Figure S1.** GSRs values per patient for the two different breads.

Supplement: Supplementary file 1 [file nutrients-10-01964-s001.zip › Supplementary Files/Figure S1.pdf]

## Slide 1
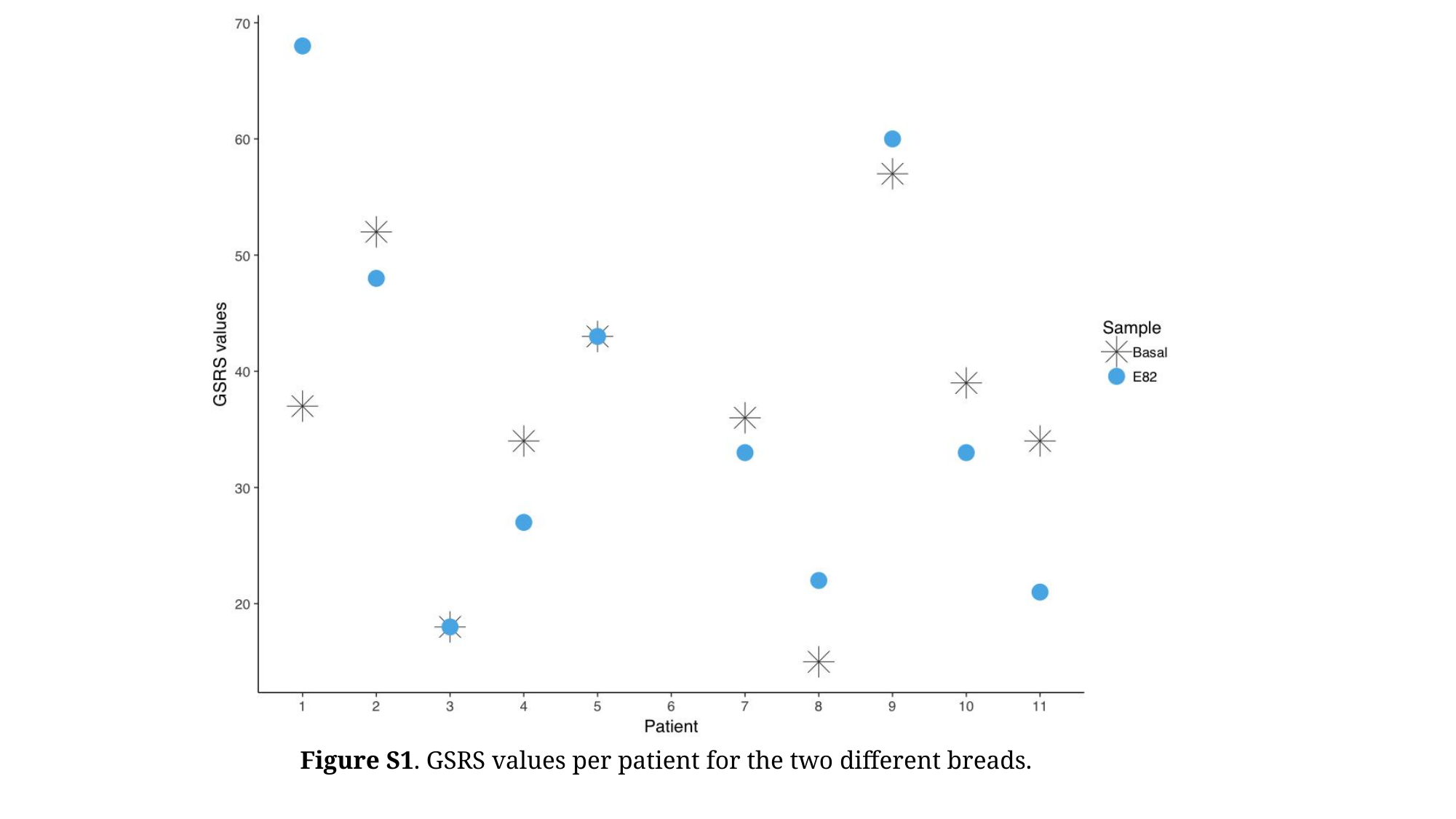

Figure S1. GSRS values per patient for the two different breads.

Supplement: Supplementary file 1 [file nutrients-10-01964-s001.zip › Supplementary Files/Figure S1.pptx]
